# Reliability and Short-Term Intra-Individual Variability of Telomere Length Measurement Using Monochrome Multiplexing Quantitative PCR

**DOI:** 10.1371/journal.pone.0025774

**Published:** 2011-09-30

**Authors:** Sangmi Kim, Dale P. Sandler, Gleta Carswell, Clarice R. Weinberg, Jack A. Taylor

**Affiliations:** 1 Epidemiology Branch, National Institute of Environmental Health Sciences, Research Triangle Park, North Carolina, United States of America; 2 Laboratory of Molecular Carcinogenesis; National Institute of Environmental Health Sciences, Research Triangle Park, North Carolina, United States of America; 3 Biostatistics Branch, National Institute of Environmental Health Sciences, Research Triangle Park, North Carolina, United States of America; Tulane University Health Sciences Center, United States of America

## Abstract

**Background:**

Studies examining the association between telomere length and cancer risk have often relied on measurement of telomere length from a single blood draw using a real-time PCR technique. We examined the reliability of telomere length measurement using sequential samples collected over a 9-month period.

**Methods and Findings:**

Relative telomere length in peripheral blood was estimated using a single tube monochrome multiplex quantitative PCR assay in blood DNA samples from 27 non-pregnant adult women (aged 35 to 74 years) collected in 7 visits over a 9-month period. A linear mixed model was used to estimate the components of variance for telomere length measurements attributed to variation among women and variation between time points within women. Mean telomere length measurement at any single visit was not significantly different from the average of 7 visits. Plates had a significant systematic influence on telomere length measurements, although measurements between different plates were highly correlated. After controlling for plate effects, 64% of the remaining variance was estimated to be accounted for by variance due to subject. Variance explained by time of visit within a subject was minor, contributing 5% of the remaining variance.

**Conclusion:**

Our data demonstrate good short-term reliability of telomere length measurement using blood from a single draw. However, the existence of technical variability, particularly plate effects, reinforces the need for technical replicates and balancing of case and control samples across plates.

## Introduction

Telomeres are non-coding repetitive DNA sequences (TTAGGG) at the end of chromosomes, and play important roles in maintaining genomic integrity and stability [1]. In dividing cells telomeres progressively shorten and, in response to short telomeres, cells normally undergo replicative senescence [2]. Senescent cells increase in aging tissues [3], [4]. While it remains unclear whether accumulation of senescent cells adversely affects tissue function [4]–[6], telomere length has been increasingly examined as an indicator of aging and age-related diseases including diabetes [7], [8], cardiovascular disease [9], [10] and cancer [11]–[15]. A real-time PCR technique, which is inexpensive, high throughput, and requires only a small amount of DNA (∼10 ng), has made the measurement of telomere length widely available in epidemiologic studies [16].

In most of these studies, an individual's telomere length is estimated from a single blood draw. Even within a short period of time, however, an individuals' measurement may vary because of both biological and technical reasons and thus imprecision inherent in a single measurement could influence estimates of disease association. Using multiple measurements averaged across different time points could provide more precise determinations for individuals than a single measurement, but obtaining multiple measurements is often impractical in large epidemiologic studies.

Few published studies have addressed the reliability of telomere length measurements, particularly over shorter time period, whereas long-term data show that telomeres generally shorten over the course of a decade or more [17]–[19]. The present study was carried out to examine the reliability of a single measurement of telomere length in sequential blood samples collected over a 9-month period and to evaluate the relative magnitude of different sources of variation in telomere length measurements, determined using a single-tube, monochrome multiplex qPCR method [20].

## Results

Results of telomere length measurements are summarized by visit in Table 1. Mean telomere length measurement at any single visit was not significantly different from the average of the other 6 visits (all paired t-tests *p*>0.05). Mean telomere length measurement appeared to be slightly smaller at visit 4; however, such a trend was not continued at the following visits and there was no clear indication suggesting greater similarity in telomere length measurements of proximal visits versus among measurements of distal visits. Also, there was no seasonal variation in telomere length measurements.

**Table 1:** Mean telomere length measurements by visit (N = 24)^a^.

	January-March				May	July	October	
	Visit 1	Visit 2	Visit 3	Visit 4	Visit 5	Visit 6	Visit 7	Average
Mean	1.23	1.24	1.22	1.19	1.25	1.21	1.23	1.22
Range (min- Max)	(0.72–1.77)	(0.80–1.87)	(0.74–1.85)	(0.76–1.86)	(0.80–1.95)	(0.73–1.90)	(0.74–2.12)	(0.76–1.89)
P-value^b^	0.82	0.46	0.99	0.09	0.16	0.25	0.66	

a Out of 27, 24 subjects had telomere length measurements at all 7 visits.

b Paired t-test between mean telomere length measurement at a single visit vs. average of the other 6 visits.

**Figure 1:**
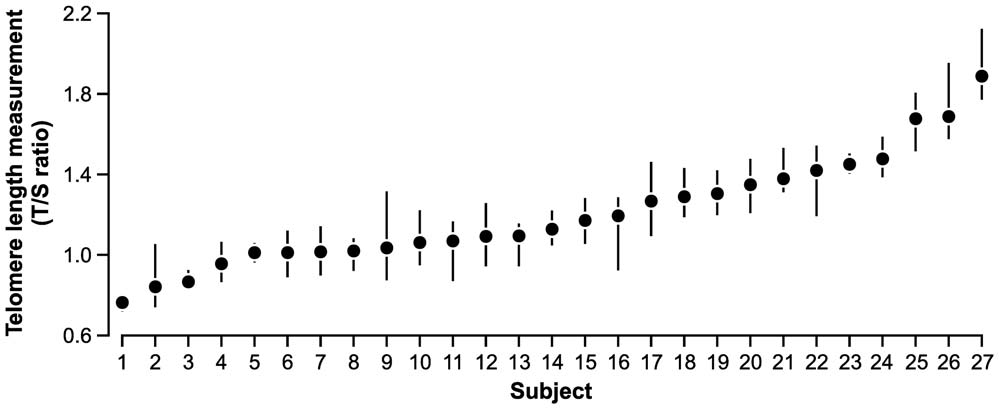
Range of variation in relative telomere length measurements for 27 participants. The dots indicate mean relative telomere length for each individual and the vertical bars indicate the range of values. Results for the 27 women are presented in rank order for individual mean values.

**Figure 2:**
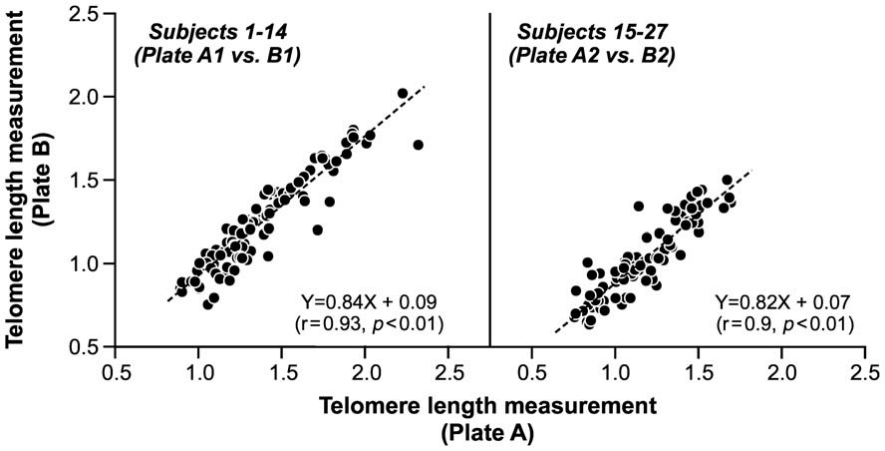
Scatterplots of mean relative telomere length measurements estimated from duplicate plates. Each dot in the scatterplot represents mean relative telomere length measurements from a single blood draw with the x-value representing the average value from plate A (A1 or A2) and the y-value representing the average value from plate B (B1 or B2), which are the replicate plates of plate A1 and A2. All samples from subjects 1–14 were analyzed in plates A1 and B1, and those from subjects 15–27 were analyzed in plates A2 and B2.

The range of variation in telomere length measurements is illustrated by subject in Figure 1. The variation in telomere length measurements was separated into variance components for subject (between-subject), visit, and residual variance using the linear mixed model described in Methods. The model was adjusted for plate-to-plate variability, as our data showed systematic differences in telomere length measurements from different PCR plates (Figure 2). After controlling for plate effect, the intraclass correlation coefficient (ICC), which is the ratio of the between-subject variance to the total variance, was estimated to be 64%. Within subject, the variance due to visit was minor, contributing about 5% of the remaining variance. The ICC ranged between 55% and 72% in different subgroups (Table 2). In the present study, each plate also included controls that are determined to have high, medium and low T/S ratio values. We performed a sensitivity analysis to assess whether inclusion of the control data in the analysis influenced the results. Even though the estimated effect of plate slightly changed, the estimates of between-subject variability were virtually unchanged, from 64% without considering the controls to 63% with inclusion of the control data in the analysis.

**Table 2:** Variance components for telomere length measurements estimated from linear mixed model.

		Var(between subject)		var(subject-visit)		Residual	
	No.	σ^2^ (SE) a	% total variance	σ^2^ (SE) a	% total variance	σ^2^ (SE) a	% total variance
All women	27	37.8 (10.5)	63.8	2.9 (0.7)	4.9	18.6 (0.9)	31.3
Age <45 y	10	51.2 (23.2)	72.1	2.4 (1.0)	3.4	17.3 (1.3)	24.5
Age ≥ 45 y	17	30.0 (10.6)	57.4	3.2 (0.9)	6.1	19.0 (1.1)	36.5
Premenopause	16	38.1 (13.7)	64.0	3.0 (0.9)	5.0	18.4 (1.1)	31.0
Postmenopause	11	35.4 (15.4)	63.0	2.9 (1.0)	5.1	18.0 (1.3)	32.0
White	16	29.9 (10.9)	57.4	2.5 (0.9)	4.8	19.7 (1.2)	37.8
African American	11	40.9 (17.9)	66.9	3.5 (5.7)	5.7	16.7 (1.2)	27.4

a Values shown are the estimates X 1000 for easier reading.

**Figure 3:**
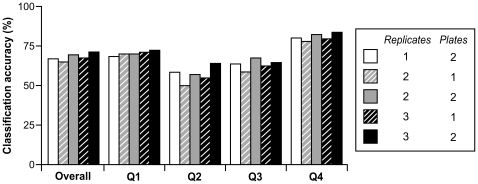
Classification accuracy of measurements from a single visit versus average of multiple visits. Telomere length measurement at any single visit was estimated using a different number of technical replicates (indicated as different color bars), and compared against the “gold standard”, which was the average of telomere length measurements estimated from 6 technical replicates at multiple visits (7 visits in 24 women and 6 visits in 3 women). Women are classified into quartiles (Q1, Q2, Q3 and Q4) using the gold standard value. Individual bars for women in each quartile represent the percentage of the time women are correctly placed in the gold standard quartile using different numbers of replicates.

In epidemiologic studies of biomarkers without established cut points, measurements are often categorized based on the distribution in the study population to examine the association with an outcome of interest. We therefore examined classification accuracy of telomere length measurement at a single visit considering average telomere length measurement across 7 visits over 9 months as the “gold standard”. We first categorized 27 women into quartiles based on telomere length measurements averaged across 7 visits over 9 months (1st quartile: <1.016; 2nd quartile: 1.016-<1.127; 3rd quartile: 1.128-<1.378; and 4th quartile: ≥1.378), and compared the “gold standard” quartile classification with quartile placement based on telomere length measurement at any single visit for each woman, using cutpoints appropriate to the respective visit number. Overall, there was relatively good agreement in quartile classification of telomere length measurement between a single visit and the gold standard. In 133 out of 186 (72%) visit-based comparisons, women were placed in the same quartile category as they were in the gold standard classification (Figure 3). Among the remaining 28% that were not in agreement, none of them were off by more than 2 quartile categories.

We then explored the impact of number of technical replicates or plates on agreement of a single visit with the gold standard. Each colored bar in Figure 3 represents a different number of technical replicates used to estimate telomere length measurement at a single visit. For example, the white bars represent results based on sampling a single replicate from each of the three replicates on each of two plates for each visit and each woman. The overall agreement between results using measurement at a single visit and the average of multiple visits decreased gradually with decreasing number of technical replicates used to estimate telomere length measurement at a single visit: from 72% with 6 technical replicates to 70% with 4 replicates and 65% with 2 replicates. Regardless of the number of technical replicates used for estimation of telomere length at a single visit, quartile agreement (i.e., placing women in the same quartile) was better for women whose gold standard was in the first and 4th quartiles compared to those in the middle quartiles. This tendency was particularly evident with fewer technical replicates, indicating that increasing number of technical replicates was particularly effective in improving classification accuracy in women whose telomere length was in the middle 50% in the distribution. We also found that with the same number of technical replicates, the classification accuracy tended to be slightly better when technical replicates were used from duplicate plates rather from a single plate.

## Discussion

There has been great interest in telomere length in blood cells as a potential biomarker of cancer risk and other various aging-related phenotypes. Because many epidemiologic investigations involving telomere length rely on a one-time measurement of telomere length, reliability of the measurement over time should be established.

Using blood DNA samples from 27 women collected in 7 visits over a 9-month period, the present study reported a relatively high intraclass correlation coefficient of 0.64, suggesting that telomere length measurement at a single time point is a good representation of an individual's telomere length within a short period of time.

Telomere length measurement in blood cells might be influenced by a transient shift in the distribution of blood cell subpopulations caused by infection or acute inflammation, as blood cells comprise heterogeneous subpopulations with different replicative history and telomerase activity [21]. The present data did not directly address the question, but suggests that such influence, if any, is far exceeded by between-individual variability in telomere length measurements.

Part of the variability in telomere length measurements can be attributed to the variation inherent in any quantitative PCR method [22]. In our study, relative telomere length was determined using a recently-introduced single-tube, monochrome multiplexing quantitative PCR, which reduces pipetting-induced variation in telomere length measurements compared to the previously described qPCR method where telomeres and single copy genes are amplified independently in separate tubes that are usually located on separate plates [20]. Even using the single tube method, we still observe appreciable plate-to-plate variability. To decrease the effect of inter-plate variability on the association between telomere length and outcomes of interest, it would be ideal to analyze all samples on a single plate, but even with 384 or newer high density 1,536 well plates, most epidemiologic studies will require multiple plates. The influence of possible differential misclassification bias can be minimized both by ensuring balance of cases and controls within plates and by placing technical replicates of samples on multiple plates. Inclusion of technical replicates within and across plates not only improves precision of an individual's measurement, but also allows investigators to generate reliable results even when an individual qPCR reaction fails [23].

Our analysis of quartile classification agreement between telomere length measurement at a single visit vs. the “gold standard” (i.e., average of multiple visits over a 9-month period) further supports the short-term reliability of telomere length measurement. It also provided a new way of examining the influences of the number and plate assignment of technical replicates on the classification accuracy of telomere length measurement at a single visit. Given that classification accuracy of women in the first and 4^th^ quartiles is less subject to number of technical replicates compared to those in the middle quartiles, the disease association estimates for a comparison between the longest versus shortest quartiles of telomere length seem unlikely to be affected by poor precision of telomere length measurements. On the other hand, fewer number of technical replicates and the resulting loss of precision will lead to misclassification of subjects with telomere length in the middle categories, obscuring possible dose-response relationships between telomere length and the disease of interest.

Some aspects of our current investigation may limit the generalizability of our findings. First, our study is based on a small set of adult women volunteers. Although we observed a similar magnitude of good intraclass correlation coefficients for telomere length measurements within different subgroups by age, race and menopausal status, our findings may be less generalizable to children or male populations. For example, there have been reports suggesting accelerated telomere shortening in children [24], males [25] and African Americans [26] compared to adults, females and whites, respectively. Additionally, in this study we adopted a recently-introduced single-tube, monochrome multiplexing quantitative PCR [20] for determination of telomere length. Despite increased efficiency in time and source DNA over the previously described method, this new multiplex qPCR might require more extensive optimization to be widely utilized [27].

In summary, our data demonstrated an overall good reliability of telomere length measurement within 1 year. However, some level of technical variability and significant plate-to-plate variation found in this study suggest the importance of technical replicates and balanced allocation of affected and unaffected samples within a single plate.

## Materials and Methods

### Ethics statement

All samples were collected anonymously from adult volunteers for use in laboratory assay evaluation. Identifying information such as name and contact information was temporarily maintained and was destroyed at completion of the last specimen collection. All participating women were provided a copy of the consent form and a staff member and witness signed that the consent was explained and agreed to by the subject. The study including the consent procedure described was approved by the Institutional Review Board of the National Institute of Environmental Health Sciences, NIH and the Copernicus Group Institutional Review Board.

### Study subjects

The Sister Study (www.sisterstudy.org) is a national prospective cohort study to investigate environmental and genetic risk factors for breast cancer and other end points in 50,884 women who had a sister diagnosed with breast cancer. As a part of the Sister Study, a series of quality control samples from anonymous donors was collected using a standard collection protocol to assess short-term and seasonal variability in various analytes and to determine if study measures are sufficiently stable over time to justify analysis using samples from a single time point. Twenty-eight non-pregnant adult women were recruited from the Raleigh/Durham area of North Carolina through local advertisements, and they provided blood and urine specimens at up to seven different visits. Four visits were scheduled every two weeks over the course of two months and three subsequent visits were scheduled at three month intervals over a 9-month period. At each visit, all participants completed a questionnaire to provide information on hours of sleep, alcohol use, cigarette smoking, and medications taken in the past 24 hours. Out of 28 women, one missed 3 visits and was excluded from this analysis. The present analysis included 24 women who completed all 7 scheduled visits and 3 women who missed only one visit, totaling 186 visits from 27 women (16 whites and 11 African Americans) with age ranging from 35 to 74 years (mean: 47.8 years).

### Quantification of telomere length

Genomic DNA was extracted from 5 mL of stored frozen packed cells using the QIAamp DNA Blood Maxi Kit (Qiagen, Valencia, CA). Extracted DNA was eluted in TE buffer and stored at −20°C following quantification using Quant-iT™ PicoGreen dsDNA reagent (Invitrogen). For each sample, 10 ng of DNA were aliquoted into 3 wells on duplicate 384-well plates, providing a total of 6 technical replicates parsed across 2 plates. Due to the capacity of the 384-well plate, all longitudinal samples from subjects 1–14 were quantified in plates A1 and B1, and those from subjects 15–27 were measured in plates A2 and B2.

Telomere length was determined as the relative ratio of telomere repeat copy number to a single copy gene copy number (T/S ratio) using the monochrome multiplex quantitative PCR protocol described by Cawthon [20]. The T/S ratios have been shown to be proportional to the classical Southern blot on terminal restriction fragments [16], [28]. Telomere primer sequences were telg (ACACTAAGGTTTGGGTTTGGGTTTGGGTTTGGGTTAGTGT), telc (TGTTAGGTATCCCTATCCCTATCCCTATCCCTATCCCTAACA), and albumin was used as the single copy gene reference using primers modified with the addition of a 5′ = GC clamp to shift melting temperature: albu (CGGCGGCGGGCGGCGCGGGCTGGGCGGaaatgctgcacagaatccttg) and albd (GCCCGGCCCGCCGCGCCCGTCCCGCCGgaaaagcatggtcgcctgtt). The reagent components and final concentrations were 900 nM each primer (IDT), 1X AmpliTaq Buffer II (ABI), 3 mM MgCl_2_, 0.2 mM per dNTP, 1 mM DTT, 1 M betaine, 0.75X SYBR Green I and 0.625 U AmpliTag Gold polymerase.

A 5-point standard curve made from a set of pooled DNA samples was included for each assay plate using a 2.5 fold dilution series of DNA amounts ranging from 1.9 to 75 ng. Each plate also contained six technical replicates of each of three controls previously determined to have high, medium and low ratio values. Plates were run on a BioRad CFX384 with the following cycling parameters: 95°C for 15 minutes; 2 cycles at 94°C for 15 seconds, 49°C for 15 seconds; 33 cycles at 94°C for 15 seconds, 62°C for 10 seconds, 74°C for 15 seconds, 84°C for 10 seconds, 88°C for 15 seconds. Signal acquisition at 74°C allowed for collection of the telomere Ct values, while acquisition at 88°C provided the albumin Ct values. The BioRad CFX Manager software was used to estimate DNA amounts for each sample T (telomere) and S (albumin single copy) from their respective standard curves. Standard curve efficiencies for both primer sets were above 90%, and correlation coefficients were ≥0.99. The within-plate and between-plates % coefficient of variation (%CV), which is based on the ratio of the standard deviation across replicates to the mean, were 18% and 7%, respectively. For study samples, the within-plate %CV ranged from 8.5 to 15.1%.

### Statistical analyses

Possible systematic effects of time ordering were assessed by carrying out paired t tests comparing each subject's visit-specific estimate with their mean across the other 6 visits. Pearson's correlation coefficient and linear regression were used to evaluate the relationship between telomere length measurements from duplicate plates.

A linear mixed model was used to estimate the components of variance for each telomere length measurement associated with variation among subjects and variation between time points within subject as below.
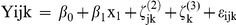
where *β_0_* is the overall mean, *ζ^(3)^_k_* is a random effect for subject k, which captures the deviation of the subject's mean from overall mean (*k* = 1∼27). *ζ^(2)^_jk_* is a random interaction between time point and subject (j = 1∼7). This random effect takes on a different value for each subject and time point combination, and because it captures the variability across time within the subject, it is nested within subjects [29]. The *i* subscript (*i* = 1∼6) indexes the ith technical replicate, which is nested within subject and visit number. An additional fixed effect (*x_1_*) was added in the model to accommodate any systematic effect of plate (l = 1∼4) on measurements. The intraclass correlation coefficient (ICC) was calculated as the proportion of total variance due to subjects. Telomere length measurements were slightly skewed to the right, and therefore, natural-log-transformed values were used. The modeling was done using Stata's multilevel mixed-effects linear regression procedure (xtimixed module, Stata 10.0).
